# Making particularity travel: Trust and citizen science data in Swedish environmental governance

**DOI:** 10.1177/03063127221085241

**Published:** 2022-04-05

**Authors:** Dick Kasperowski, Niclas Hagen

**Affiliations:** Department of Philosophy, Linguistics and Theory of Science, University of Gothenburg, Gothenburg, Sweden

**Keywords:** particularity, citizen science, trust, environmental governance, Artportalen, actor-network theory

## Abstract

This paper focuses on how particularities are performed and made to travel through the creation of trust. The Swedish Species Observation System (Artportalen) is one of the largest inscription and calculation centers for citizen data in the world, used extensively by public authorities in Sweden. Observations by members of the public become actionable through environmental governance laws in Sweden. These observations are made through networks of things and humans in which trust is created but unevenly distributed. Important for them to be trusted and to travel are such things as computer software to filter and map observations, red lists, GIS-tools to determine time and place, and validation committees. However, trust is more concentrated in a core set of actors, and there depends on interpersonal relations – though these relations are facilitated by other parts of the epistemic system.

According to a standard tale, scientific knowledge connects words and matter. Knowledge, meanwhile, can travel, sometimes extensively, in time and space. This is commonly described as achieved through scientific rigor, which raises knowledge to a level of relative universality beyond particular observations. In Science and Technology Studies (STS), such universality is usually seen as achieved through the circulation of particularities in the extension and strengthening of networks (Latour, 1999). It is therefore a result of translations: the extensions and strengthening of networks *do* universality, as knowledge does not travel on its own but is translated between actors.

Managing particularity is part of creating universality (Latour, 1993; O’Connell, 1993; Timmermans and Berg, 1997), and it seems inescapably intertwined with and travels along universal knowledge. Particularities are often articulated in scientific or technical controversies and institutional inadequacies. Caught in the universal standards of an ‘abstract system’ (Giddens, 1990) makes travel of particularity difficult (Moreira, 2012). However, sometimes particularity is called for.

How, then, is particularity done and made to travel? This article focuses on particularities as they are performed and made to travel through the creation of trust. To illustrate this, we investigate how the gap between matter and word is overcome, as observations by members of the public are made to travel and through trust become actionable in environmental governance in Sweden. Relying on conceptual resources from Actor-Network Theory (ANT) and from studies of the distribution of work and trust in epistemic cultures, our point of departure is that trust in a particularity is performed and contingent. We ask two sets of questions: How is trust distributed, formed and stabilized to extend and strengthen particular observations to travel and become actionable? Where do entities meet and interact in this practice, and what are important access points?

For this study, we turned to a large on-line inscription device and center of calculation (see below) called Artportalen (https://www.artportalen.se), and the use of this facility by civil servants at Swedish regional public authorities who are responsible for environmental governance. During the last two decades, Artportalen has developed from a report system and a calculation center established by amateur ornithologists into an advanced inscription device for public decision-making and knowledge on environmental sustainability, biological change and degradation in Sweden (Liljeblad, 2021; Lundquist, 2018).

Empirical qualitative data for this study comprises eighteen semi-structured in-depth interviews and follow-up interviews with civil servants at the Swedish Forest Agency (SFA), the Regional County Boards (RCB) in Sweden, and at the Swedish Agricultural University in workshops with developers and scientists at Artportalen. The interviews, follow-ups and workshops were conducted during late autumn and winter 2019, and in the spring and autumn 2020.

## Some conceptual resources

### The travel of particularities

We argue that particularity gains epistemic and practical value in the extension and strengthening of networks of things and humans. Specifically, we are interested in how particularity becomes representational and is preserved by the circulation of trust assigned to or removed from different actors and materialities in a network at hand (Knorr-Cetina, 1999, 2007; O’Connell, 1993; Shrum et al., 2001). To accept a particular observation as representing (in our case) a fact of nature, entities are formed and stabilized to extend and strengthen it, and transformations are employed to preserve its particularity and to make it travel.

ANT has highlighted the power produced through delegation to non-human actors, resisting analytical asymmetries between humans and non-humans. Standardization studies have been developed from such sensitivities and have increasingly displayed how standards are embedded and enabled through human trust. This does not imply a departure from ANT general symmetry: When analyzing the performativity of trust in the travels of observations, we can remain agnostic about the distribution of agency and its normative ramifications.

In this study, we extend conceptual resources from ANT, usually concerned with the perspective of the scientists (Latour, 1995), to the actors and materialities involved in environmental governance based on citizen observations. We focus on those actors and their technologies at hand, assigned with the task to evaluate whether a particular observation can be trusted and made to travel in the process of Swedish environmental governance.

### Chains of translations and concentration

The turning of an observation into a fact happens in chains of transformations and by circulating references, usually with a scientist as the originator (Latour, 1995). Throughout the process of observing, measuring and sampling, locality and particularity are lost through shifting materialities and continuity, gaining compatibility, standardization, text, calculation, circulation, and relative universality. Thus the gap between matter (in our case, observation) and word (red list, laws) is overcome, as particular observations are made to travel (in environmental governance).

Chains of transformation, in the distribution of human and non-human entities, which make citizen observations useable in the exercise of public authority, might be distributed differently. They need not feature linearity, where every inscription refers to others that come before it and that is itself transformed by the next inscriptions. We suggest that the referential quality of a particular observation and its ability to travel through a network for public authority is dependent on how trust is circulated as links in chains are created.

The more, and the shorter or closer, the links between a particular observation and those who make judgments about those links, the more robust those judgments may be. In the search for conceptual resources to describe the travel of particularities as an effect of how trust is distributed and accumulated in relations, we would like to apply the concept of concentration in this study. This concept denotes how gaps are managed by the partial stabilization of a network through the trust that is developed, accumulated and invested in human and non-human entities to make a particularity travel.

### Trust: Individuals and abstract systems

Since the early days of STS, trust has been an important issue in understanding the production of scientific knowledge. Preserving particularity and making it travel, on our understanding, is attained by calling upon different material and human entities in creating trust. Partly following Moreira (2012) and Strathern (2002), we understand trust as an action performed on the entities at hand in a dynamic relationship between the particular and the universal. The possibilities of travel on behalf of the particular are performances acted out in a network.

Trust is not created evenly in the network. Entities form into networks, and the strength of network relations waxes and wanes. Furthermore, stability changes as entities in the network can be assigned trust differently to make particularity travel or, conversely, be distrusted to end travel altogether.

Many STS scholars emphasize the trust in science that derives from interpersonal relations, familiarity and face-to-face interaction (e.g. Hedgecoe, 2012; Shapin, 1994: 414). In this, they might be seen as criticizing perspectives that locate trust in systems (e.g. Giddens, 1990; Luhmann, 2000).

We might expect to identify system-based trust in studies of epistemic cultures that analyze the distributed character of scientific work and minimize the individual as an epistemic subject (Knorr-Cetina, 1999, 2007). We also might expect to find system-level trust in studies of reliance and dependence on technologies and other black-boxes in the distribution of work in research (Shrum et al., 2001: 682), with no one individual being an expert, or even fully proficient, in all the considerations which inform decisions (Brown et al., 2016: 89). Responsibility and authority are decentralized, and thus trust is found on an abstract level. Systems and interpersonal encounters are interfoliated to create bases of trust (Möllering, 2001).

In this article we look at interpersonal trust as part of and developing out of systems. Giddens (1990) considers face-to-face interactions and commitments at ‘access-points’ (p. 83) of abstract systems (Brown et al., 2016, 89). An access point is where actors meet and interact with an abstract system, that is, instances and entities that call upon some kind of action on behalf of actors. This is where trust is tested, established and maintained – or lost – and where the abstract system, according to Giddens (1990), is especially vulnerable. In the case of Artportalen, there are several access points available for actors that include, but are not limited to, observation protocols, GIS maps and filters (see below).

Inspired by Knorr-Cetina and Giddens, we suggest that in large distributed network of this study trust as being created dynamically, becoming more or less concentrated in ‘access-points’, as particular observations are made to travel.

## Artportalen: From amateur calculation to inscription center in public decision-making

Before the development and implementation of Artportalen in 2000, civil servants at the SFAs and RCBs in Sweden were largely dependent on local databases. These comprised data from local inventories, compiled by employees or hired consultants; these data also could include observations made by members of local birding clubs and from individuals who had demonstrated a local expertise in flora and fauna. Many of the local offices of the RCB and SFA also had their own repositories of inventories and relied heavily on local knowledge among civil servants and employees. Many of the observers reporting to the early version of Artportalen also worked as civil servants at the RCB or SFA. Once a year, individuals and clubs were summoned to their local RCB office to voluntarily provide observational data to the public authority.

In the beginning, Artportalen was not intended as an instrument for environmental governance. A gradual change was initiated by civil servants at one of the offices of the RCB. They successfully argued that it was absurd to have several parallel and local systems; instead, they wanted a system with the infrastructural potential to become standardized and for all users. With funds from the Swedish Environmental Protection Agency (SEPA), Artportalen was developed to become a standardized and simplified way for users to report observations (civil servant, RCB).

The initial focus on birds has been expanded: some other vertebrates, and also some insects, plants and fungi. However, this expansion has not corresponded with the establishment of validating committees for judging the quality of all observations, with birds remaining the most developed taxonomic kind.

With the development and implementation of Artportalen, practices of the protection and management of the Swedish natural environment have undergone significant changes, becoming highly distributed and standardized beyond the earlier local repositories.

Artportalen still serves the interests of different amateur communities, such as bird watchers, as the system also offers ranking lists (Lundquist, 2018: 81). However, Artportalen supports a highly distributed epistemic community of interested members of the public, scientists, consulting and research companies that report observations on many different species.

Currently, Artportalen is one of the largest inscription and calculation centers in the world concerning nature, conservation and environmental issues. It encompasses more than 80 million observations and is indispensable in creating aggregated data for the Swedish Species Information Center (SSIC) located at the Swedish University of Agricultural Sciences. The SSIS has been stabilized over the 30 years it has been in place, producing aggregated data on biodiversity in Sweden. An important task for SSIC is the calculation of data from Artportalen to prepare to make decisions on listing species as endangered or threatened, which involves a distributed network of experts, including professional scientists and lay experts, to determine the status of species for red listing (SLU, 2020).

The relation between the aggregated data and individual observations reported through Artportalen is vital for the exercise of public authority in environmental governance in Sweden. The SSIC would not have been able to develop into an obligatory passage point for knowledge and decision-making without the efforts of many actors, the humans and non-humans making up the capacity of Artportalen, to make particularities travel and to create aggregated data.

In STS, centers of calculation have largely been conceptualized in terms of scientific knowledge production, ranging from local laboratories to large-scale conglomerates associated with big science. In this respect, centers of calculation can be highly distributed, but still exclusively within networks of professional actors. In the large-scale citizen science project that is investigated in this study, the center of calculation is differently distributed, as it is based on public participation for the production of data. Thus, SSIC as a center of calculation depends on inclusive functions and mass mobilization by the Swedish public, where anyone can inscribe data (observations).

However, not all observations are equal in terms of certainty and the actions they might entail. This affects the capacity of observations to travel. In ANT terminology, what we encounter in our case is neither the chains of transformations present in classical studies of science in action (Latour, 1999), nor what has more recently been presented as chains of obligations (Latour, 2010), but a combination of these two processes. The successful or failed combination of these chains depends on how trust is circulated over time in the network. The observed species in question or the combination of several species, validation committees, the red lists, and the technologies available for determining the place and time of observations, etc. It is a combination of entities that can make an observation travel to meet the law that makes it actionable in environmental governance.

To explicate these processes, we now turn to our empirical data to understand the distribution of human and non-human entities in more detail, as used by civil servants at the RCB and the SFA.

## Responsibilities of the civil servant: Connecting dots and laws

Several entities are at the disposal of a civil servant for connecting dots on the digital maps with laws for environmental governance in Sweden. A civil servant with the right to exercise public authority is charged with the task of implementing official government environmental policies through the different laws at hand. In Sweden, these include the Endangered Species Protection Ordnance and The Habitats Directive, for which one needs observations. Being a civil servant responsible for the protection and management of the Swedish natural environment at a regional level includes managing many different cases and requests concerning the environment. These include providing advice on larger interventions, such as deforestation by large forest companies or individual forest owners, the establishment of natural reserves, evaluations of the impact on nature in large infrastructural projects, building permits, the planning of roads, etc. Responsibilities also include addressing minor concerns, for example, a consultation with a private landowner if an old fragile oak tree should be preserved. Accordingly, the duties of a civil servant at RCB or SFA include supervision, consultation and the issuing of permits.

This demands that the civil servant determine what observations have been made and which ones are more or less authoritative. Several of our informants stress that these demands have increased with the implementation of Artportalen and the Endangered Species Protection Ordnance:

The laws need data and observations about the occurrence of species. Without observations, you cannot implement the laws. Assessments of observations are hard to do; they are complex. In the end, it’s up to the court to decide. But it is a new role for the civil servant to interpret how the laws should be implemented, for instance, the Endangered Species Protection Ordnance. There is access to more observations all the time, and more demands on the Regional County Board, as it is the authority responsible for species and habitat protection …. It is also a question of resources. (civil servant, SFA)

With these laws in place, particular observations of red-listed species have to be worked upon if they are to travel and connect with the legal space that makes them actionable for decision-making in planning or for trial in one of the environmental courts. However, informants stress a discrepancy between observations and the law:

If we were to follow the species protection ordinance by the letter, we would have to close Sweden down. The species to be protected are clearly listed in the Endangered Species Protection Ordnance. But how should this be done? Many ‘noes’ can mean changes in the law. Law and observation have a certain discrepancy. Artportalen is a reporting tool; the law is something else. (civil servant, RCB)

To determine what has been observed, there must be a mutual concern for both the law and the species in question. The quote above observes not only the species to be preserved, but also the law. This relation between the law and the observation is attained by managing and coordinating several entities in making a particular observation travel. However, the entities called upon might be more or less trusted and, therefore, assembled differently in the networks of materialities and humans that are accessible to the civil servant.

## Observations through entities: Filters, lists, time, place, species, and validation

### Filtering data

A typical case starts with the civil servant at their desk, gazing at a computer screen. A matter of concern, an ‘access point’, has presented itself and prompts action from the RCB or the SFA. The screen, if opened up from one of the digital resources at hand, will produce a map where observations that are reported via Artportalen to the SSIC are visible as dots. Some of these dots represent observations of red-listed species. The primary function of these filtered access points is to determine whether an observation is one that prompts further action on behalf of the civil servant and whether or not it will start to travel.

The civil servant then uses different filter functions to determine if the observations are ones that will prompt further action. Because there is more data than ever, the different filter functions have been developed to help manage the data deluge that users are confronted with when turning to Artportalen and the SSIC: ‘Filters were introduced to not get drowned in observation of species that are not protected by law. We have too much data, and filters are there to make our work more effective’ (civil servant, SFA).

In some cases, a dot signifies a sensitive observation that is not publicly visible. Observations of very sensitive species collected by clubs or individuals are sometimes not reported or, if reported, are not tagged with correct GPS-coordinates in Artportalen, to keep their locations unknown. In cases where sensitive observations are reported, the filter and the WebGis will signal that the civil servant needs to contact a colleague with a higher authorization at the RCB or the SFA to access the observation in SSIC.

### Red lists

The filter functionalities in the GIS-tools make it possible for civil servants to quickly determine whether the observation is of a red-listed species and if so, what category it belongs to: ‘It is primarily red-listed species that prompt action’ (civil servant, RCB). An important aspect of the particular observation and the possibility of its traveling is thus the relation it has to the aggregated data presenting the general knowledge of the status of different species in Sweden:

The Habitats Directive increases the need for observational data. Data has a much more significant role now. Invasive species. The Species Protection Ordnance and The Habitats Directive list species that are to be protected at the EU-level as well – this has major consequences for our work. The red list is very important. (civil servant, RCB)

### Time and place

One task for the civil servant is to determine where and when an observation was made, to pinpoint a particular location for it, important for it to move further in the network. GPS-coordinates and a photo (taken by the observer and attached to the Artportalen report protocol) best allow the observation to be put in relation to others in the biotope or in the vicinity. Place, though, has to be combined with time. It might be more difficult for older observations to travel, but that also depends on the species observed and who made the observation. An exact time and location strongly help an observation to travel, but are not always necessary, as other entities can be used to support it.

### Species

The species has to be determined as a stationary or transient kind. A bird might be passing by; an observation of a bird fifteen years ago is very unreliable. The civil servant is then assigned to certify such observation, often by contacting the observer in person.

Some species’ connection to a specific time and place are more significant for the observation to travel. An observation of a red-listed plant or a moss might be particularly important, as might a combination of several observations of a relatively stationary animal. Whether an observation of a species will travel may also depend on its relation to other species. This is the case for observations of ‘signaling species’, associated with a habitat or environment that is sensitive and needs protection and will probably also hold other (not yet observed) endangered species.

### Validation

All observations in Artportalen undergo validation, which takes place in several stages. The first validation is made by the observer who must be report the observation according to Artportalen’s protocol; the observer tags the information about time and place and preferably includes a photo or video. If the observer is uncertain about information, they can also mark the observation as such. The next stage takes place when the observation is available for other observers and their comments: ‘The visibility of observations to others on Artportalen creates a “threshold” for the quality of observations. There are few observations that are wrongly reported’ (developer, Artportalen). The last stage is when certain observations (of either red-listed or very rare species in Sweden) are chosen for further evaluation by one of the eighteen validation committees. In this last instance, the taxonomic affiliation is evaluated to certify the location and that the observation is really of the species in question.

The committees have full access to all the observations reported to Artportalen and are responsible for determining the status of the observation; thus, they are at least formally important if observations are to travel.

If all stages of validation are conducted, this process results in an observation receiving a higher status in Artportalen, which will, in turn, affect its possibility of traveling. A large part of the validation process relies on personal interaction and the status of the observer. We now turn to how these entities are formed and stabilized to extend and strengthen trust in particular observations, so that they can travel while legal obligations can be met.

## Extending and strengthening observations: Interpersonal relations and the concentration of trust

### Accessing the observation

Let us revisit the civil servant at their desk, gazing at a computer screen, producing a map on which observations are visible as dots. The first connection between the observation and the red list is delegated to the digital resources at hand; only species that are red-listed will be ‘filtered out’. At this point, the civil servant might find that the observation is not fully accessible for purposes of action. During the entire process, issues of trust and (to a lesser extent) distrust, relying on personal interaction, are played out.

There is a firm principle of transparency and trust guiding Artportalen, and all observations and reported information are openly accessible and free for use by anyone. Because of this access, if the observation is of a red-listed species that is especially sensitive, the exact location of the observation, the species and the observer’s name are not disclosed, as mentioned above. For example, observations of large birds of prey are not always accompanied with clear positioning coordinates – to avoid disturbing the birds by keeping locations of nesting places secret. Experts at The Swedish University of Agricultural Sciences and the validation committees decide upon this classification. The ‘full’ observation is then only accessible to the observer, members of the validation committee and to a few selected officials at each RCB.

The filter then, depending on the sensitivity of the observation, will only show the observation in a ‘diffused form’, not disclosing the exact position of the species in question. If this is the case, the civil servant has to gain permission from the higher-ranking official to access the observation, according to the Swedish Public Access to Information and Secrecy Act (2009: 400). No details on the species, location and time of the observation, or the name of the observer, are disclosed to the civil servant at this stage.

Upon request, access to the observation is granted. It is seldom denied, unless the higher-ranking official deems the observation to lack importance for the task at hand. For instance, it might be outside the area of concern, or very old, etc. Once access to the observation is granted, the civil servant also keeps these rights for handling of future cases, until the higher-ranking official decides otherwise. Access to protected observations should be strictly limited to the civil servants who need it for their work (civil servant, RCB).

Now, it is the civil servant’s task to further test if the connection between observation and the red list can be trusted for additional, perhaps, legal actions. To do this, the dot is ‘opened up’ in Artportalen to check the status of the observation, the time and location thereof, to check whether there is a photo attached, by whom the observation was made and to decide if that person can be trusted.

### Enlisting as an observer: Submitting to practices of trust

If a volunteer is interested in adding observations to Artportalen, he or she must submit them under certain clearly formulated conditions. Artportalen has important requirements for trust that are to be established with potential observers through interpersonal relations. One cannot submit observations to Artportalen anonymously. Observations of species have to be reported according to its protocols and its obligatory inputs: time, location, species, and observer.

An observer must provide their name, address, phone number, e-mail address and may provide a brief description of their interests. The e-mail address must be functional so that administrators at Artportalen can contact the observer. False names or handles are a sign of mistrust and, in such cases, administrators have the right to terminate accounts. The emphasis on trust and accessibility is aptly described by one of the developers of Artportalen: ‘Observations are perishable; therefore, you need to be able to access those that enlist as observers. They have to always be available’ (developer, Artportalen).

Photos or other types of media that are linked to or uploaded according to the observation protocol must depict the individual, the place of the observation, tracks, litter or nest. Administrators at Artportalen have the right to remove images that do not live up to these criteria. All observations reported to Artportalen are subject to some form of validation by other users, experts at the Swedish Agricultural University or validation committees. This might result in changes of the status of the observation. All such changes are stored in the Artportalen protocol and cannot be erased by the observer or anyone else.

### Accessing the observer: Trust to travel for the local

To access and interact with the observer is our informants’ single most mentioned and emphasized example of how trust in an observation is created.

Personal knowledge of individual observers, if he or she is employed by a consultancy firm or maybe an employee at another branch of the RCB. You go to the source, the observer or the colleagues to help you do an assessment of when, where and who. It helps if you know if a certain employee at a consultancy firm is responsible for the observation or if it is a member of a local club. (civil servant, RCB)

According to informants, the standardized and distributed use of Artportalen has actually increased the possibilities and need for personal interaction with the observer. It is particularly through the access point of the protocol, used by observers to report sightings of species and by validation committees and civil servants to access information on the observer, that trust is tested, potentially established and maintained – or lost. As such, the report protocol features an instance of concentrated resources for establishing trust, affecting an observation and its potential to travel in the network.

Trust in the observer and the observation is established by several things. Several of our informants highlight personal knowledge of who the observer is and how well that individual has created observations in Artportalen over time. Trust has temporality:

If you are uncertain, for instance, about the species in question, you end up with the individual person – someone who has a history of good documentation and tagging of observations. You get to know the person who is reliable. If you then see the name in other observations, you can trust that person and his or her documentation of observations. (civil servant, RCB)

A record of ‘good’ observations is also relying on the local or regional environmental knowledge performed by the observer. The civil servants often ‘contact local ornithologists or clubs to investigate the quality of an observation’ (civil servant, SFA). Local knowledge is coupled with local connections, for instance, if the observer is a member of a local association.

If you state membership in an association or club, being an employee in a consultancy firm contracted by the RCB, trust in the observation increases. (civil servant, SFA).

During the twenty years that Artportalen has been in use, it has developed into a highly distributed and standardized system for environmental governance in Sweden. This has also contributed to, and further facilitated, personal interactions and access to the individual observer for creating trust in observations. Informants repeatedly point to the shared knowledge of who you can trust as an observer, held in the network of the branches of RCBs. Before Artportalen, there was only local and largely tacit knowledge among the civil servants at RCB, but now it is shared beyond the local, facilitating both a distributed validation of observations, potentially among all observers present on Artportalen, as well as personal interaction in creating trust to make particular observations travel. The introduction and development of Artportalen has produced a new ecology of participation by engaging and valuing localized observers, but also making their observations possible objects for all participants to validate, trust or distrust. This draws attention to both the temporalities and localities of trust and travel in observations (Krzywoszynska et al., 2018).

### Species and validation committees: To be stationary is to travel

Civil servants at RCB or the SFA not only stress the importance of the validation process, but also that different validation committees are in place, as they have been functioning before Artportalen became an instrument for environmental governance. This is the case with the evaluation committee on birds, which has been in place since Svalan [The Swallow] was established in 2000 as a digital reporting system for amateur ornithologists. Svalan has been integrated in Artportalen since 2015. However, for environmental governance observations birds might not be as important as those of plants and other more stationary species: ‘Stationary species, cryptogams, fungi or vascular plants are the most important for evaluating the value of a particular locality, maybe to be proposed for a natural reserve’ (civil servant, SFA). However, evaluation committees for other species groups are not as well developed as those for birds, making the traveling of observations a more complicated process in these groups. This can even mean that civil servants have to visit the place of the observation to make an evaluation and close the gap between the particular observation and the evaluation committee.

Observations are sometimes not that exact; positions are not exact. The observer doesn’t give the exact location or the observation can be misplaced …. In some cases, I visit the place myself, if the species or area in question is important. If there is some kind of uncertainty in those cases, you have to check for yourself sometimes. (civil servant, SFA)

The Swedish Agricultural University, which hosts Artportalen and SSIC, is concerned with the problem on the uneven status of validation committees. In the development of standards for validation in Artportalen, National Validation Groups (NVGs) have been established. These include representatives from the Swedish Agricultural University, Artportalen, non-profit environmental organizations, other expert committees, museums, etc. The NVGs are responsible for developing a ‘sound validation culture’ and assigning members to distinct species groups. In some cases, civil servants from the RCB or the SFA are members of validation committees. Some observations of species, in this context, travel more easily than others. However, evaluation committees in 2017 consisted only of very few experts, seldom more than 5–6 individuals, and were not as well developed in species groups for fungi (2 individuals), lichen (1), moss (4), spiders (1), dragonflies (1) and amphibians (2) in comparison to vascular plants (62), and birds (238). To create the trust necessary for an observation of a stationary species that is not a nesting bird, the civil servant might have to interact with the observer, an expert in the validation ‘committee’, an employee in a consultancy firm, a colleague or with a trusted contact in a local bird-watching club. In some cases, one person can encompass all these capacities or roles, closing the gap between the particular observation and the evaluation committee. In the absence of a developed validation committee, trust is increasingly created via interaction in a core set of individuals (Collins, 1981). To be stationary is therefore to travel, but not without extensive personal interaction in creating trust.

## Discussion

Artportalen exhibits large-scale distribution with approximately 15,000 observers and 250,000 visits yearly, producing environmental data in Sweden and resulting in the aggregated data that underpin the Red List Index. It is a process in which individual actors are minimized as epistemic subjects, and where trust is developed and valued through the rigor of statistical conventions. However, the epistemic culture also values the individual trusted observer for performing particular observations. In this epistemic culture, accessibility and visibility of observers are highly valued and important (see Weigelhofer and Pölz, 2016). The report protocol of Artportalen features many of the functionalities that Giddens recognizes in his ‘access points’. Here, actors actually meet and interact with the system. This is where trust is tested, established and maintained – or lost (Giddens, 1990). More detailed studies on access points in different forms of citizen science are needed to understand where in the research process the citizen is granted access and how trust is developed or lost.

In the development of Artportalen, according to our informants, the more standardized and developed the system has become, the more it has both facilitated and created a need for interpersonal trust. Civil servants’ accounts of their practices are narratives about performing observations through personal interactions for creating trust; they constantly refer to trust in explaining how observations are managed.

An inscription and calculation center such as Artportalen, compensates not only for interpersonal interaction, but also relies upon and facilitates such interactions. Artportalen is an example of how the extensions and strengthening of networks of humans and materialities are needed to *do* particularity and make it travel. However, particularities are not made to travel without universalism, in this case, to meet both the aggregated data of red lists and the universality of Swedish law.

### Distrust and environmental activism: Toward a core set?

Our informants seldom bring up issues of distrust; when they do, it is in the context of environmental activism. For instance, observations submitted by individuals and non-governmental organizations with the intent of stopping logging in valued forest areas might not travel further than the desk and computer of the civil servant.

For example, if someone has just recently registered an account and reports doubtful observations to stop logging – this must be investigated. It is important to be objective. If you see a badly tagged observation, and that person comes up several times, you become suspicious. (civil servant, SFA)

One informant addresses this issue in the context of less developed evaluation committees.

Amphibians are not well validated. Some time ago, a lot of observations of water salamanders were reported by unknown observers; you get suspicious. Sometimes people report observations to stop building and exploitation plans. (civil servant, RCB)

The validation of observations and the reliance on face-to-face interaction for creating trust suggest that using Artportalen in environmental activism may tend to generate distrust. Making observations travel toward legal settings is a performativity, accessible to a select trusted few who can create a ‘technoscientific normativity’ – that systematically excludes others as untrustworthy (Bora, 2010: 3).

This is an issue that needs more attention – exploring how the trusted observer as an epistemic subject develops over time. Earlier in this article, we suggested that the referential quality of an observation and its ability to travel through a network for public authority were dependent on the quality of chains of translations. The more, shorter or closer steps there are between the particular observation and those who make judgments about them, the more robust those judgments will be. In the organizational structure of SFA and RCB in Sweden, the circulation of trust to make an observation travel in the direction of the red list and the laws is done in a relatively small network. In spite of a highly distributed network of humans and materialities, the concentration of trust mostly occurs in a close-knit network relying on interpersonal knowledge, familiarity and face-to-face interaction.

Informants’ accounts present us with a highly distributed system that relies on a close-knit network of actors resembling the notion of a ‘core set’. This concept usually refers to the group of scientists involved in the eventual resolution of a given technical controversy (Collins, 1981). Following suggestions that core sets, especially at ‘science/public interfaces, are, in fact constituted from “non-technical” issues – political, ethical, economic’ (Michael and Birke, 1994: 81), data in this study also point to trust as an important aspect forming a core set beyond or outside science, simultaneously contributing both to scientific knowledge and environmental governance.

### Contexts of trust

Finally, comparisons between different national contexts of using citizen data for environmental governance must be carried out, bearing in mind that these display trust in societal institutions and science differently. Sweden usually scores high in international comparisons on surveys of public trust and confidence between individual citizens (Martinson and Andersson, 2020). It has been argued that Nordic countries are rated as highest in the world when it comes to interpersonal trust; however, a recent dip may be discernible in Sweden (Holmberg and Rothstein, 2020). According to national surveys, 75%–85% of the public has, since the mid 1980s, expressed ‘fairly or very high’ trust in science and researchers (Bergman and Bohlin, 2020). Thus, the issue is if there are better and worse suited contexts for citizen science and the use for citizen data in sustainable governance, then this study points to trust as an important component to be further explored.

## Conclusion

To make observations travel to meet the legal space that makes them actionable for decision-making in a highly distributed network of human and non-human entities, trust formed over time in personal interactions is necessary. Highly standardized formats for collecting and processing data, such as Artportalen, associated with expansive abstract systems both need and facilitate the importance of interpersonal relationships in a core set for creating trust in making observations travel.

In conclusion, results from this study suggest that analyzing trust in large distributed networks of citizen science would benefit from understanding trust as created dynamically, becoming more or less concentrated, forming inclusion or exclusion, in certain access points, as particular observations are made to travel or not. Such analyzes would raise further questions about the role of the politics of trust in societal institutions and science (Frow, 2020).
